# Evaluating the Efficacy and Safety of Botulinum Toxin in Treating Overactive Bladder in the Elderly: A Meta-Analysis with Trial Sequential Analysis of Randomized Controlled Trials

**DOI:** 10.3390/toxins16110484

**Published:** 2024-11-08

**Authors:** Yu-Hsuan Chen, Jen-Hao Kuo, Yen-Ta Huang, Pei-Chun Lai, Yin-Chien Ou, Yu-Ching Lin

**Affiliations:** 1 Education Center, National Cheng Kung University Hospital, College of Medicine, National Cheng Kung University, Tainan 701, Taiwan; chenyuhsuan064@gmail.com (Y.-H.C.); thomas42913@gmail.com (J.-H.K.); debbie0613.lai@gmail.com (P.-C.L.); 2 Department of Surgery, National Cheng Kung University Hospital, College of Medicine, National Cheng Kung University, Tainan 701, Taiwan; uncleda.huang@gmail.com; 3 Department of Urology, National Cheng Kung University Hospital, College of Medicine, National Cheng Kung University, Tainan 701, Taiwan; 4 Department of Physical Medicine and Rehabilitation, National Cheng Kung University Hospital, College of Medicine, National Cheng Kung University, Tainan 701, Taiwan

**Keywords:** overactive bladder, elderly, botulinum toxin type A, systematic review, trial sequential analysis

## Abstract

Overactive bladder (OAB) significantly impairs quality of life in the elderly. Although the intradetrusor injection of botulinum toxin type A (BoNT-A) is a treatment option, its effects on older adults remain uncertain. This study aimed to evaluate the efficacy and safety of BoNT-A intradetrusor injections in elderly OAB patients through a systematic review and meta-analysis. A comprehensive literature search was conducted using the PubMed, Embase, Cochrane Library, Scopus, and CINAHL databases from inception to 30 May 2024. The primary outcomes were improvements in daily urinary incontinence (UI) episodes and patient-reported outcomes, while the secondary outcomes focused on potential adverse events. Four randomized controlled trials with 803 participants were included. BoNT-A injections significantly reduced daily UI episodes at 4–6 weeks (mean difference [MD]: −3.82; 95% confidence interval [CI]: −6.29 to −1.35) and at 12 weeks (MD: −2.17; 95% CI: −3.22 to −1.12). However, BoNT-A was associated with an increased risk of elevated post-void residual (Risk Difference [RD]: 0.154; 95% CI: 0.058 to 0.251) and urinary tract infection (RD: 0.111; 95% CI: 0.005 to 0.217), with no significant difference observed in the initiation of catheterization or hematuria. Trial sequential analysis confirmed a sufficient sample size and statistical power. In conclusion, while BoNT-A effectively manages OAB in the elderly, careful post-injection monitoring is warranted due to its potential risks.

## 1. Introduction

Overactive bladder (OAB) is a chronic syndrome that affects both men and women [1]. The International Continence Society defines it as a condition characterized by urinary urgency, with or without urgency urinary incontinence, usually accompanied by increased daytime frequency and nocturia, in the absence of infection or another underlying pathology [2,3]. These bothersome symptoms significantly impact patients’ quality of life [1,4]. The prevalence of OAB increases with age, making it a particularly concerning issue in the aging population [5].

The management of OAB typically begins with behavioral therapy or pharmacological treatments, which are considered first-line options [6]. However, less than half of patients achieve satisfactory outcomes with these initial approaches [7]. Antimuscarinic agents, including solifenacin, darifenacin, and fesoterodine, have been shown to relieve OAB symptoms with minimal cognitive impact and few central nervous system side effects in elderly patients after short-term use [8,9]. However, the potential prolonged use of these agents, particularly the cumulative effects of anticholinergic burden, has raised concerns about cognitive decline in the elderly population [10,11]. Moreover, studies on oxybutynin, another antimuscarinic, have demonstrated more concerning results regarding its impairing effects on cognitive function [12]. β3-adrenoceptor agonists such as mirabegron and vibegron have been found to be effective and well tolerated in older adults [13,14]. However, most clinical trial participants were relatively healthy and lacked uncontrolled cardiovascular conditions, leaving questions about the long-term use of these medications in the elderly. Given these concerns, alternative treatments, such as botulinum toxin intradetrusor injection, have emerged as potential options for managing OAB.

Botulinum toxin intradetrusor injection is a well-established treatment for OAB [15]. Among the various types of botulinum toxins, botulinum toxin type A (BoNT-A) is the most commonly used due to its longer therapeutic duration [16,17]. In studies involving BoNT-A bladder injections, onabotulinumtoxinA (Botox^®^, Allergan, Irvine, CA, USA) was the most frequently used commercial form, whereas abobotulinumtoxinA (Dysport^®^, Ipsen, Slough, UK) was rarely utilized [18]. When injected into the detrusor muscle under cystoscopic guidance, BoNT-A is thought to act through several distinct mechanisms [19,20]. In the motor pathway, BoNT-A inhibits the release of acetylcholine at presynaptic nerve terminals in both the somatic and autonomic systems, resulting in chemical denervation of the targeted muscles [19]. On the sensory side, BoNT-A desensitizes afferent nerves by preventing the release of several neurotransmitters such as adenosine triphosphate, substance P, and calcitonin gene-related peptide [21]. Additionally, BoNT-A downregulates sensory receptors, including transient receptor potential vanilloid 1 (TRPV1), and purinergic (P2X2, P2X3) receptors, contributing to its overall therapeutic effect in managing OAB symptoms [21].

The effectiveness of BoNT-A intradetrusor injection has been demonstrated in patients with OAB who have not responded adequately to, or have experienced intolerable side effects from, first-line pharmacological therapies, such as antimuscarinic agents and β3-adrenoceptor agonists. Recent studies have also shown its superior therapeutic effects over oral medications [22]. However, few studies have specifically addressed the unique needs and responses of the geriatric population [18,23]. This gap in research highlights the need for more focused investigation into the safety and efficacy of BoNT-A in this demographic. The purpose of this systematic review and meta-analysis is to evaluate the current literature regarding its efficacy in symptom improvement and its safety profile in this vulnerable population.

## 2. Results

### 2.1. Literature Search and Study Selection

**Figure 1:**
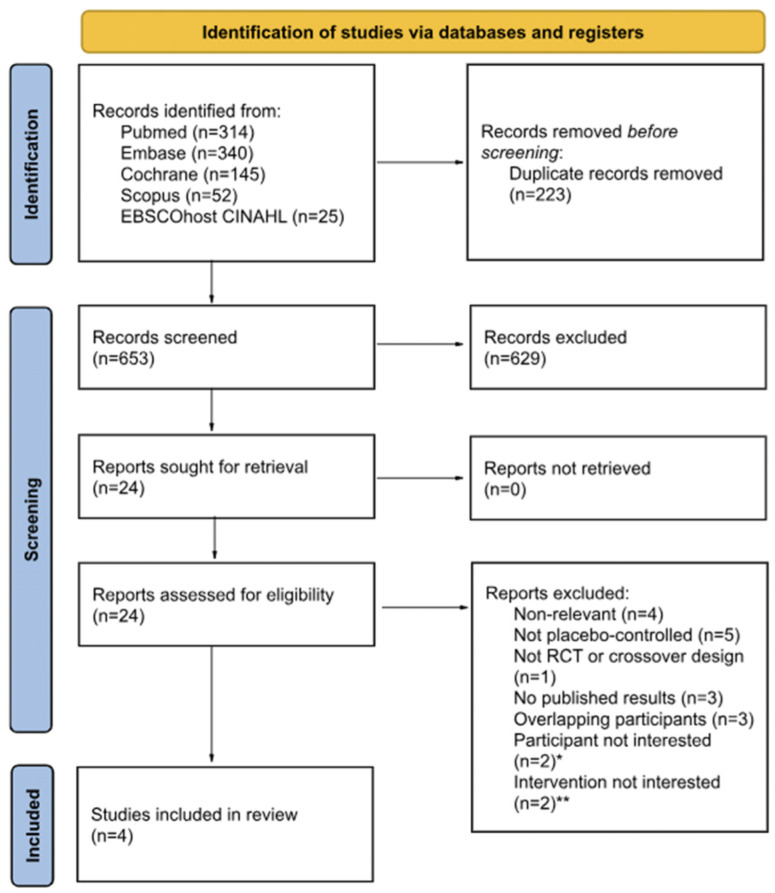
The PRISMA flow chart. PRISMA, Preferred Reporting Items for Systematic Reviews and Meta-Analysis; RCT, randomized controlled trials; CINAHL, Cumulative Index to Nursing and Allied Health Literature; Embase, Excerpta Medica Database. * Individuals who were not specifically patients with overactive bladder; ** studies where botulinum toxin was not administered via direct injection into the detrusor muscle, but rather, through alternative methods, such as instillation.

A total of 876 articles published from database inception to May 2024 were retrieved by searching PubMed, Embase, Cochrane Library, Scopus, and EBSCOhost CINAHL. Among the 653 articles retained after the exclusion of duplicate articles (*n* = 223), 629 articles were deemed unsuitable based on title and abstract reviews. A detailed examination of the full texts of the 24 remaining articles resulted in the exclusion of 20 studies. Ultimately, four studies involving a total of 803 patients were included in the systematic review and meta-analysis, along with trial sequential analysis (TSA). The flow of the literature throughout the assessment process is illustrated in a PRISMA flow chart (Figure 1).

### 2.2. Study Characteristics

For the original review, four studies were identified in the literature search (Table 1), all of which were randomized controlled trials (RCTs). These RCTs involved a total of 803 patients, all diagnosed with OAB, with the exclusion of those with neurogenic detrusor overactivity. All the involved patients received onabotulinumtoxinA (Botox^®^, Allergan, Irvine, CA, USA) bladder injections to treat their OAB symptoms. All these RCTs had participants with a mean age over 65, except for the study by Moore et al. [24]; therefore, only the subgroup of participants over 65 years old was included from this study.

### 2.3. Risk of Bias Assessment

Table 2 outlines the findings of the risk of bias assessment, conducted using the Cochrane Risk of Bias Tool 2 for RCTs. Three RCTs [24,25,27] were found to have some concerns regarding the randomization process and missing outcome data due to unclear allocation concealment and the potential impact of missing outcome data. One study [27] was flagged for some concerns related to selective reporting bias, as the outcomes were insufficiently analyzed due to an inadequate sample size. In terms of overall risk of bias, one study [26] was evaluated as having a low risk of bias, while the remaining three [24,25,27] were considered to have a high risk of bias.

### 2.4. Daily Urinary Incontinence (UI) Episodes

**Figure 2:**
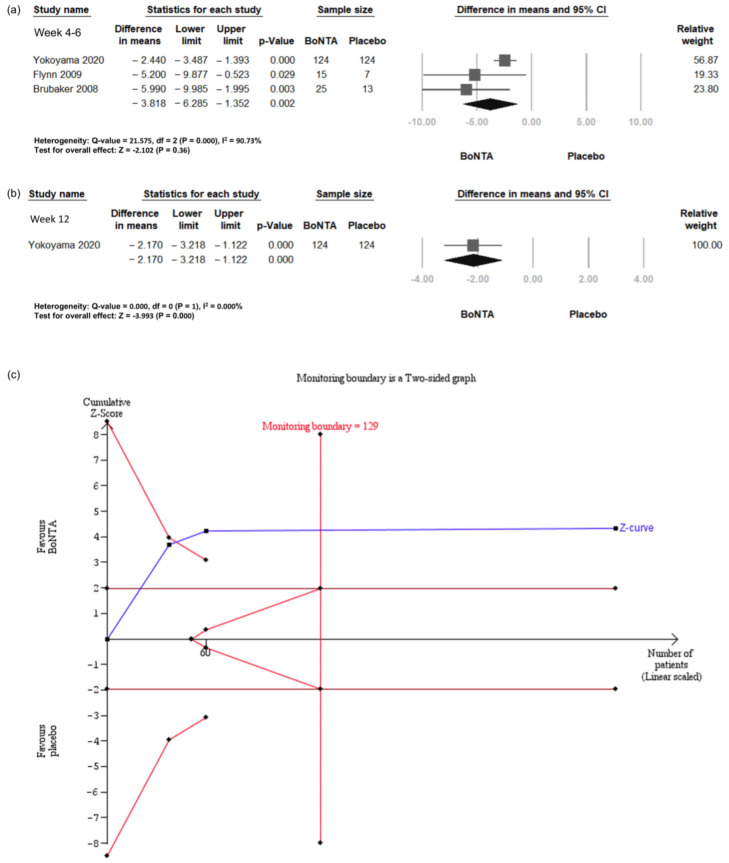
Change in daily urinary incontinence episodes in BoNT-A group and placebo group. (**a**) Forest plot for weeks 4–6. (**b**) Forest plot for week 12. (**c**) Trial sequential analysis plot. UI, urinary incontinence; BoNT-A, botulinum toxin type A; TSA, trial sequential analysis; CI, confidence interval.

The daily UI episodes were investigated in three RCTs [25,26,27], involving a total of 308 patients (Figure 2a). The meta-analysis for daily UI episodes showed a significant advantage for BoNT-A at both the 4- to 6-week and 12-week follow-up points. At 4 to 6 weeks, the mean difference (MD) indicated a significant reduction of 3.82 UI episodes per day (95% confidence interval (CI) −6.29 to −1.35, *p* = 0.002). At 12 weeks, the MD demonstrated a significant reduction of 2.17 UI episodes per day (95% CI −3.22 to −1.12, *p* = 0.000).

Trial sequential analysis (TSA) revealed that the number of enrolled patients exceeded the required information size (RIS) for comparing the daily UI episodes between the BoNT-A group and the placebo group (Figure 2b). The cumulative Z-curve exceeded both the conventional test and trial sequential monitoring boundaries for comparing the incidence of daily UI episodes between the BoNT-A group and the placebo group.

### 2.5. Patient-Reported Outcomes

**Figure 3:**
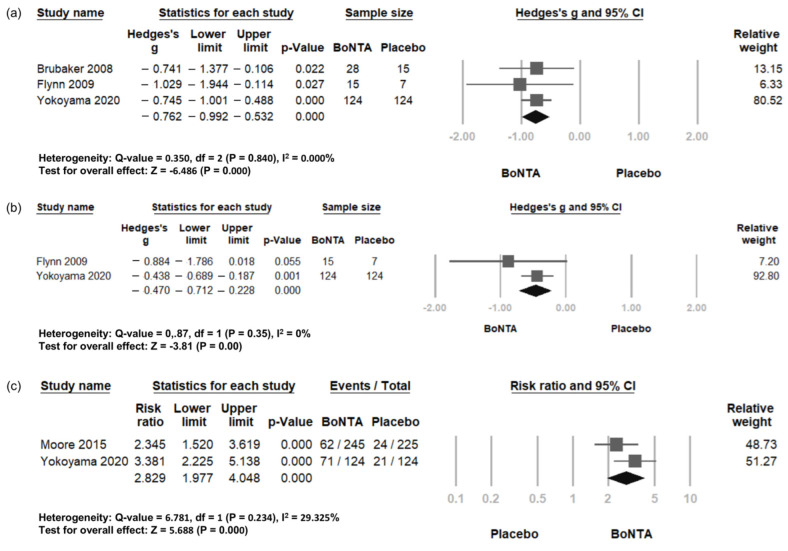
Change in patient-reported outcomes in BoNT-A group and placebo group. Forest plot of (**a**) symptom improvement, (**b**) quality of life, and (**c**) treatment-based response. QoL, quality of life; BoNT-A, botulinum toxin type A; CI, confidence interval.

Patient-reported outcomes included symptom improvement, quality of life, and treatment-based response. Three RCTs were included in the analysis of symptom improvement [25,26,27], while two RCTs were included in the assessment for quality of life [25,26] and treatment-based response [24,25]. Symptom improvement showed a more prominent effect in the BoNT-A group (standardized MD = −0.762, 95% CI −0.992 to −0.532, *p* = 0.000; Figure 3a). Quality of life improvement was also significantly greater in the BoNT-A group compared to the placebo group (standardized MD = 0.47, 95% CI −0.712 to −0.228, *p* = 0.000; Figure 3b). Additionally, the treatment-based response was also significantly higher in the BoNT-A group than in the placebo group (Risk Ratio = 2.829, 95% CI 1.977 to 4.048, *p* = 0.000; Figure 3c). Due to the variability in self-assessment items and scales across studies, TSA is not applicable.

### 2.6. Adverse Events

#### 2.6.1. Urinary Tract Infections (UTI)

**Figure 4:**
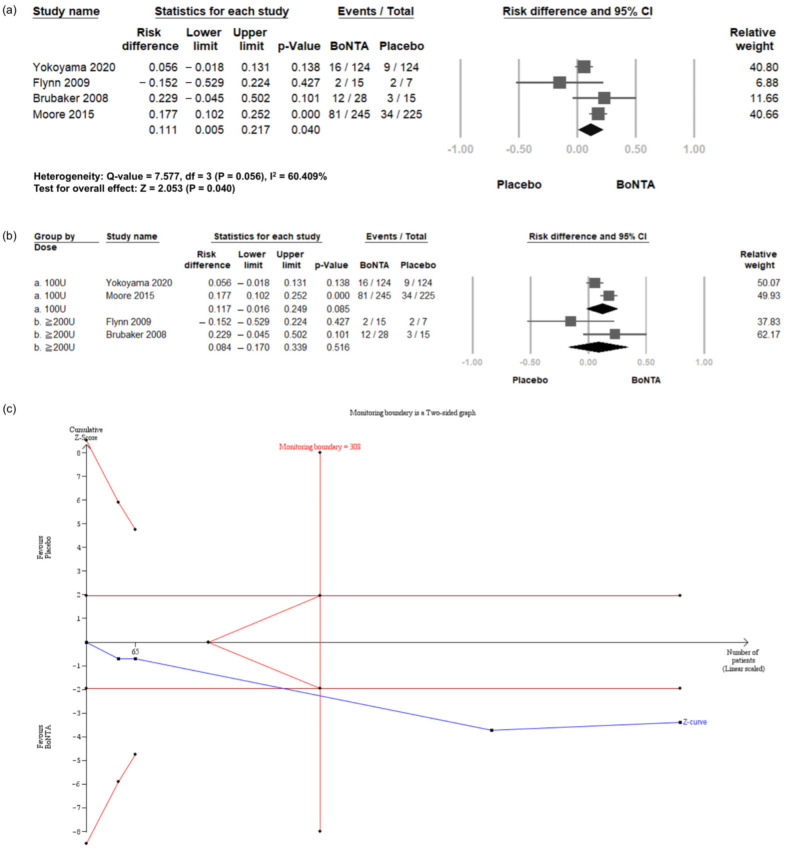
Incidence of urinary tract infection in BoNT-A group and placebo group. (**a**) Forest plot. (**b**) Subgroup analysis of 100 U and 200 U doses. (**c**) Trial sequential analysis plot. UTI, urinary tract infection; BoNT-A, botulinum toxin type A; TSA, trial sequential analysis; CI, confidence interval.

Four RCTs [24,25,26,27] reported the incidence of UTI episodes. Compared with the placebo, BoNT-A intradetrusor injections were associated with a significantly elevated risk of UTI (Risk Difference (RD) = 0.111, 95% CI = 0.005 to 0.217, *p* = 0.040; Figure 4a). These RCTs were then divided into two subgroups based on the BoNT-A dosage: 100 U or 200 U injections (Figure 4b). The results showed no significant difference for either 100 U (RD = 0.117, 95% CI = −0.016 to 0.249, *p* = 0.085) or 200 U (RD = 0.084, 95% CI = −0.170 to 0.339, *p* = 0.516) between the BoNT-A group and the placebo group.

TSA indicated that the number of enrolled patients exceeded the RIS for comparing the incidence of UTI between the BoNT-A group and the placebo group (Figure 4c). The cumulative Z-curve exceeded both the conventional test and trial sequential monitoring boundaries for comparing the incidence of UTI between the BoNT-A group and the placebo group. The TSA results reinforce the reliability of the observed UTI incidence, supporting the statistical robustness and sufficiency of the current sample size for determining clinical outcomes.

#### 2.6.2. Post-Voiding Residual (PVR)

**Figure 5:**
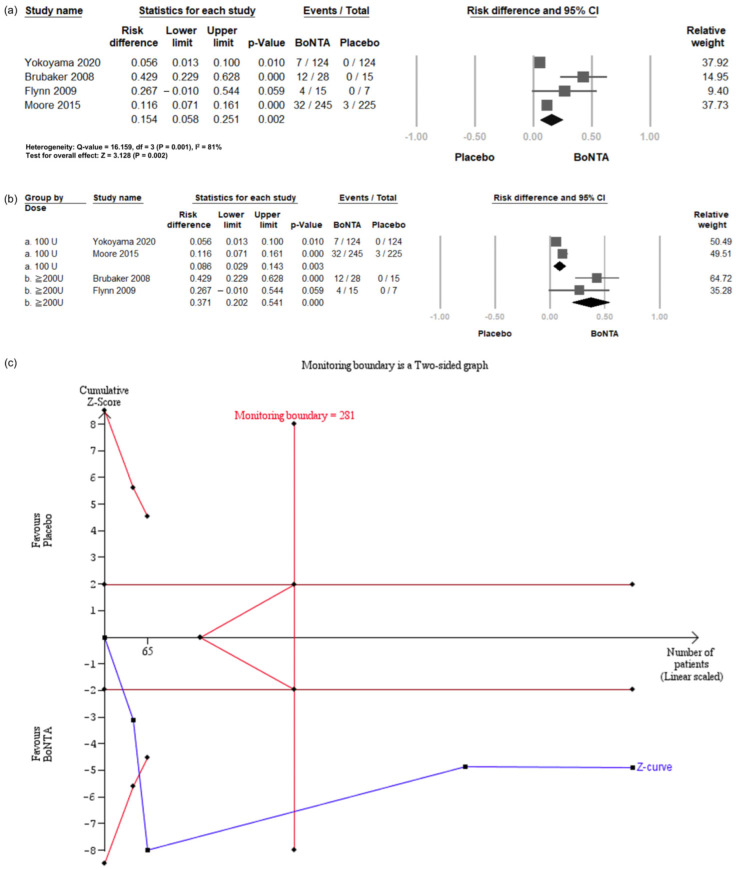
Incidence of post-voiding residual ≥ 200 mL in BoNT-A group and placebo group. (**a**) Forest plot. (**b**) Subgroup analysis of 100 U and 200 U doses. (**c**) Trial sequential analysis plot. PVR, post-voiding residual; BoNT-A, botulinum toxin type A; TSA, trial sequential analysis; CI, confidence interval.

Four RCTs [24,25,26] reported the incidence of increased PVR. In all four studies, increased PVR was defined as a volume over 200 mL. Compared with the placebo, BoNT-A intradetrusor injections were associated with a significantly elevated risk of increased PVR (RD = 0.154, 95% CI = 0.058 to 0.251, *p* = 0.002; Figure 5a). These RCTs were then divided into two subgroups based on the BoNT-A dosage: 100 U or 200 U injections (Figure 5b). The results showed a significantly increased risk for both the 100 U (RD = 0.086, 95% CI = 0.029 to 0.143, *p* = 0.003) and 200 U (RD = 0.371, 95% CI = 0.202 to 0.541, *p* = 0.000) BoNT-A groups.

TSA revealed that the number of enrolled patients exceeded the RIS for comparing the incidence of PVR over 200 mL between the BoNT-A group and the placebo group (Figure 5c). The cumulative Z-curve exceeded both the conventional test and trial sequential monitoring boundaries for comparing the incidence of PVR over 200 mL between the BoNT-A group and the placebo group.

#### 2.6.3. Initiation of Clean Intermittent Catheterization (CIC)

Four RCTs [24,25,26,27] reported the proportion of patients initiating CIC after treatment. The criteria for initiating CIC varied across these RCTs. In Yokoyama et al. [25] and Moore et al. [24], CIC was initiated in patients with PVR ≧ 350 mL, or PVR between 200 and 350 mL with associated symptoms. In Flynn et al. [26], CIC was deemed necessary if subjects complained of urinary retention symptoms and had a PVR > 100 mL. In Brubaker et al. [27], subjects with a PVR > 200 mL were instructed to start CIC.

**Figure 6:**
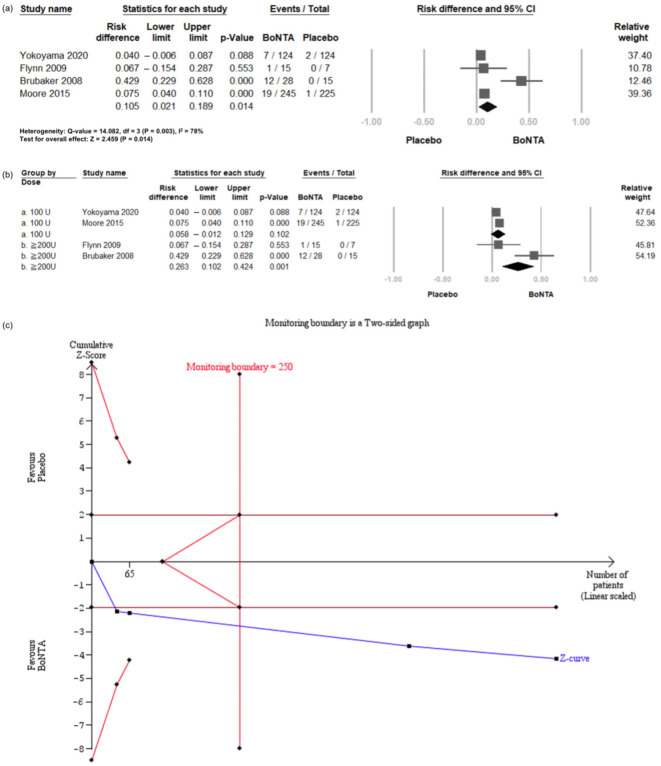
Incidence of clean intermittent catheterization in BoNT-A group and placebo group. (**a**) Forest plot. (**b**) Subgroup analysis. (**c**) Trial sequential analysis plot. CIC: clean intermittent catheterization; BoNT-A: botulinum toxin type A; TSA: trial sequential analysis; CI, confidence interval.

Compared with the placebo, BoNT-A intradetrusor injections significantly increased the risk of CIC initiation (RD = 0.105, 95% CI = 0.021 to 0.189, *p* = 0.014; Figure 6a). These RCTs were then divided into two subgroups based on the BoNT-A dosage: 100 U or 200 U injections (Figure 6b). The results showed a non-significant increase in the 100 U group (RD = 0.058, 95% CI = −0.012 to 0.129, *p* = 0.102), but a significantly elevated risk in the 200 U group (RD = 0.263, 95% CI = 0.102 to 0.424, *p* = 0.001).

TSA revealed that the number of enrolled patients exceeded the RIS for comparing the incidence of CIC between the BoNT-A and placebo groups (Figure 6c). The cumulative Z-curve exceeded both the conventional test and trial sequential monitoring boundaries for comparing the incidence of CIC over 200 mL between the BoNT-A group and the placebo group.

#### 2.6.4. Hematuria

**Figure 7:**
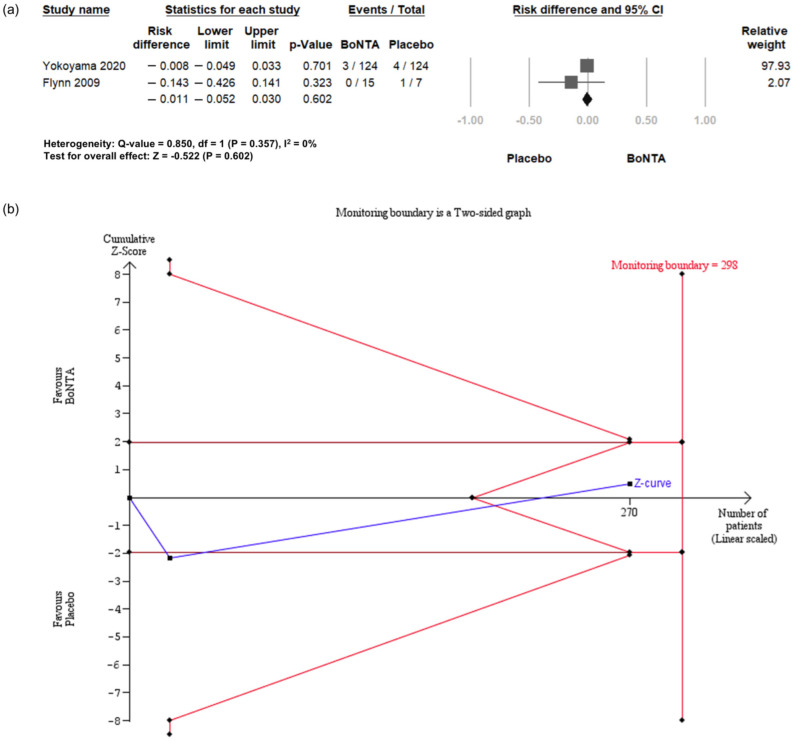
Incidence of hematuria in BoNT-A group and placebo group. (**a**) Forest plot. (**b**) Trial sequential analysis plot. BoNT-A, botulinum toxin type A; TSA, trial sequential analysis; CI, confidence interval.

Two RCTs [25,26] reported the incidence of hematuria. Compared with placebo, BoNT-A intradetrusor injections did not show a significantly elevated risk for hematuria (RD = −0.011, 95% CI = −0.052 to 0.030, *p* = 0.602; Figure 7a).

TSA indicated that only 90% (270 of 298 patients) of the RIS was accrued for comparing the incidence of hematuria between the BoNT-A group and the placebo group (Figure 7b). The cumulative Z-curve crossed the futility boundaries, leading to the conclusion that existing evidence is sufficient to show that BoNT-A intradetrusor injection is not associated with an increase in risk of hematuria.

## 3. Discussion

To the best of our knowledge, this study is the first meta-analysis of RCTs assessing the efficacy and safety of BoNT-A intradetrusor injections, specifically focusing on the elderly population with OAB. We conducted this meta-analysis using the most up-to-date evidence and TSA. To capture the effects more accurately, we analyzed different follow-up periods and dosages. Overall, the pooled analysis demonstrated a significant effect of BoNT-A in reducing UI episodes in elderly OAB patients, with the cumulative power supporting a true treatment response. Additionally, a significant improvement in self-reported assessments was observed in the BoNT-A group. Regarding adverse events, the risk of elevated PVR and UTI episodes was significantly higher in the BoNT-A group, although there was no significant difference in the incidence of hematuria between the two groups.

Previous systematic reviews have shown that BoNT-A intradetrusor injections reduce UI episodes in adult patients with OAB, with effects observed at both the 4–6-week and 12-week follow-up periods [28,29,30,31]. Our meta-analysis revealed that both BoNT-A 200 U and 100 U were superior to the placebo in reducing daily UI episodes in elderly OAB patients during the 4–6-week follow-up, and persisted for up to 12 weeks after BoNT-A intradetrusor injection. These findings support the use of BoNT-A as a viable intervention for managing UI in elderly patients, consistent with prior systematic reviews in adult populations. Despite elderly patients having more sensitive bladders at baseline compared to younger individuals, BoNT-A intradetrusor injections remain effective in controlling OAB symptoms [32,33]. In addition to its ability to induce chemo-denervation in the detrusor muscle, we believe the therapeutic efficacy of BoNT-A in addressing bladder hypersensitivity in older bladders is largely attributed to its capacity to induce a sensory blockade [21,34].

Moreover, our study found that BoNT-A doses above 100 U showed greater improvements than the placebo in the scores of symptom assessment and quality of life during follow-up from weeks 4 to 12. This could be attributed to the reduction in UI episodes, which may help prevent social isolation in the elderly, reduce anxiety and embarrassment, and improve sleep quality [35,36]. Although these subjective assessments support the use of BoNT-A intradetrusor injection in elderly OAB patients, the proportion of patients willing to undergo repeat injections indicates their satisfaction [37]. Previous observational studies have demonstrated that male patients exhibit a lower propensity for repeat treatments [38]. This is likely due to increased urethral resistance, which predisposes them to higher PVR volumes and a consequent need for CIC. Furthermore, observational studies in geriatric cohorts have reported a 42% rate of repeat injections among elderly patients, indicating inherent limitations of this therapy [39]. In other words, despite significant symptom improvement, the low rate of repeat injections suggests that side effects may affect the treatment’s acceptability. The four RCTs included in this analysis did not provide data on patients’ willingness to undergo repeated injections, highlighting a potential area for further investigation and refinement in future studies.

These findings underscore the efficacy of BoNT-A; however, its potential adverse effects must also be carefully considered. With regard to the direct chemo-denervation effect on the detrusor muscle, elevated PVR and urinary retention are frequent concerns following BoNT-A intradetrusor injections [28]. Previous systematic reviews have shown that PVR significantly increases after BoNT-A intradetrusor treatment in adult OAB patients [29,30,40]. Nonetheless, CIC is typically a temporary requirement with a relatively low incidence [41]. A retrospective study indicated that only 1.8% of patients receiving BoNT-A intradetrusor injection required CIC for more than 12 weeks [42]. Our study further demonstrated a higher occurrence of significant PVR in the elderly following BoNT-A intradetrusor injection, with 9.5% of patients in the BoNT-A group requiring CIC. Notably, Liao and Kuo’s study suggested that frailty, rather than chronological age, was a key factor associated with increased PVR after intradetrusor injection [43]. Moreover, Arrom et al. noted that age was not a significant predictor of adverse events in multivariate analyses [44]. Our study also demonstrated that adverse events, such as elevated PVR volumes and the need for CIC, were more frequently observed with the 200 U dosage, while the incidence of CIC in the 100 U BoNT-A intradetrusor injection subgroup showed only a trend without reaching statistical significance. Although our study did not identify a single conclusive predictive factor, earlier studies imply that urinary retention may result from diabetic effects on detrusor contractility or bladder outlet obstruction due to prostatic enlargement in male patients [45,46,47]. Given that retention is a potential outcome, patients should be adequately informed about the possible need to manage CIC or use an indwelling catheter prior to undergoing BoNT-A intradetrusor injections. Additionally, for the elderly population, starting with a 100 U injection is a more prudent choice.

UTI remains a frequent yet challenging issue following intradetrusor BoNT-A administration [48]. A recent systematic review reported a 29.8% prevalence of UTI post-BoNT-A intradetrusor injection for managing OAB [49]. Our research revealed a notably elevated incidence of UTIs in the BoNT-A group. Factors such as storage and voiding dysfunction have been proposed as contributors to recurrent UTI [50,51]. This may be attributed to reduced bladder compliance and heightened detrusor pressure, particularly in older individuals [52]. A bladder with compromised compliance results in elevated intravesical pressure during both the filling and voiding stages, leading to ischemic changes in the bladder [53,54]. The chemo-denervation effect of BoNT-A intradetrusor injections can relax the detrusor muscle, reduce bladder storage pressure, and improve bladder compliance, theoretically lowering the risk of UTIs caused by high bladder pressure [55]. However, in practice, BoNT-A intradetrusor injections in elderly patients have been associated with an increased incidence of UTIs, suggesting that other mechanisms may play a more dominant role in contributing to infection risk. Aside from improving bladder storage function, the chemo-denervation effect also reduces detrusor voiding contractility, which leads to incomplete bladder emptying and increased PVR, creating a favorable environment for bacterial growth. Elevated PVR is a well-known risk factor for UTIs, as residual urine serves as a breeding ground for pathogens [41].

Beyond the issues related to bladder storage and voiding function, host factors also play a critical role in the development of UTIs in the elderly population. With aging, there is a natural decline in the immune response, reducing the body’s ability to effectively fight infections [56]. Comorbidities such as diabetes and chronic kidney disease can further weaken systemic immune defenses, increasing susceptibility to infections [57,58]. Additionally, urothelial dysfunction, caused by both the aging process and trauma from needle injections, could be another important factor [59]. Disruption of the bladder urothelial lining can impair its ability to prevent bacterial adherence, making it easier for pathogens to colonize and invade the bladder [60].

Transient hematuria is a documented side effect after botulinum toxin injection. Potential causes may include trauma from injections, but our study found no significant difference in hematuria incidence between BoNT-A and the placebo. Previous studies also reported no significant difference [61]. Other literature exploring patients with a median age of 70 on anticoagulants reported no cases of significant hematuria [62].

There are some limitations to this study. Firstly, the relatively small sample size and lack of RCTs in this area may affect the generalizability of the findings. To address this, we employed TSA to ensure adequate statistical power and mitigate the risk of type I errors. Additionally, relying on average age rather than including all individuals over 65 may result in an incomplete reflection of the geriatric population. This limitation stems from the challenges of enrolling trials in this relatively fragile population. Despite subjective symptomatic improvement shown in BoNT-A intradetrusor injection, many actually failed to receive repeated injections due to adverse effects [33]. This study did not explore repeated injections, which may provide further insight into patient satisfaction, treatment efficacy, and adverse effects. Future prospective case–control studies may offer additional clarity on this issue.

## 4. Conclusions

This meta-analysis is the first to comprehensively evaluate and analyze the efficacy and safety of BoNT-A intradetrusor injections in elderly patients with OAB, using the latest evidence and TSA. Our analysis showed that BoNT-A significantly reduces UI episodes and improves self-reported assessments. However, there is a higher risk of large PVR volume and UTIs with BoNT-A treatment. Based on these findings, clinicians should carefully weigh the benefits of BoNT-A in controlling UI against its potential risks, particularly in elderly populations prone to infections and urinary retention. While our study offers valuable insights into BoNT-A injections for elderly patients with refractory OAB, further research is needed to establish more definitive evidence on its clinical benefits and potential risks.

## 5. Materials and Methods

This systematic review and meta-analysis was conducted according to the Preferred Reporting Items for Systematic Reviews and Meta-Analyses (PRISMA) statement [63]. The review was not registered.

### 5.1. Eligibility Criteria

We included only RCTs that recruited patients with overactive bladder (OAB) treated with BoNT-A. The inclusion criteria were as follows: (1) studies with a population with a mean age of >65; (2) patients with OAB who had not achieved satisfactory results with oral antimuscarinics or beta-3 agonists, or who were intolerant to these AEs; (3) RCTs comparing BoNT-A with a placebo. The exclusion criteria included non-randomized studies, such as case reports, reviews, descriptive studies, animal studies, or in vitro studies. 

### 5.2. Search Strategy

We conducted a comprehensive search of databases including PubMed, Embase, the Cochrane Central Register of Controlled Trials, Scopus, and CINAHL (EBSCOhost) using the keywords “overactive bladder”, “botulinum toxin”, and “elderly”, covering the period from inception to May 30th, 2024. Details of the search terms are listed in Table S1. The reference lists of the retrieved studies were also reviewed to identify additional relevant articles. No language restrictions were applied.

### 5.3. Study Selection and Data Extraction

The process of study selection was conducted by two authors (Y.-H.C. and J.-H.K.) independently. An initial screening of titles and abstracts was performed to select potentially eligible studies. After the initial screening, the full texts of the selected studies were reviewed, and the final decision was made regarding inclusion and exclusion criteria. Any discrepancies between the two authors during selection were resolved by discussion and consensus with senior authors (Y.-C.O. and Y.-C.L.). Data on participant demographics, types of intervention, dosage of BoNT-A, treatment duration, outcome measurements, and adverse events were collected. The study authors were contacted through email for further study details. 

### 5.4. Quality Assessment

The quality of the selected RCTs was evaluated using the Cochrane Risk of Bias Tool 2 for RCTs. Any disagreement was resolved through mutual discussion, and the senior authors, Y.-C.O. and Y.-C.L., made the final decision if a consensus was not reached. We summarize the risk of bias in Table 2.

### 5.5. Statistical Analysis

#### 5.5.1. Conventional Meta-Analysis

The primary outcomes included an improvement in daily UI episodes and patient-reported outcomes. The secondary outcomes comprised UTI, a PVR over 200 mL, CIC initiation, and hematuria. Changes in urinary symptoms after BoNT-A administration were analyzed as the primary outcomes using a random-effects model. Continuous outcomes, such as daily UI episodes, were reported as MD with 95% CI, while patient-reported outcomes were presented as standardized MD with 95% CI. Dichotomous outcomes, including UTI, PVR over 200 mL, CIC initiation, and hematuria, were reported as RD with 95% CIs. The I^2^ statistic was used to assess between-study heterogeneity, with cutoff values of 50% and 75% indicating low, moderate, and high heterogeneity, respectively. All meta-analyses were conducted using Comprehensive Meta-Analysis Software version 3.7 (Biostat, Englewood, NJ, USA). 

#### 5.5.2. Trial Sequential Analysis (TSA)

TSA was utilized to calculate the RIS and evaluate whether the findings were definitive [64]. We conducted TSA using software version 0.9 beta (Copenhagen Trial Unit, Centre for Clinical Intervention Research, Copenhagen, Denmark). The Biggerstaff–Tweedie (BT) method was employed to evaluate random effects. To control for type I errors in hypothesis testing, we utilized O’Brien–Fleming monitoring boundaries. The RIS was determined based on an alpha level of 0.05 (two-sided) and a beta level of 0.20, corresponding to a statistical power of 80%. The mean difference effect was estimated using a random-effects model that incorporated both the variance and heterogeneity observed among the included trials.

Statistical significance was defined by the cumulative Z-curve crossing the TSA boundaries. Specifically, an outcome was considered a true positive if the Z-curve crossed the O’Brien–Fleming boundaries before reaching the estimated RIS, or if the Z-curve surpassed 1.96 when the cumulative sample size exceeded the RIS. In contrast, if the Z-curve entered the futility zone, it was categorized as a true negative. Any analysis in which the total sample size did not achieve the RIS was regarded as underpowered.

## Figures and Tables

**Table 1:** Study characteristics of included studies. OAB, overactive bladder; RCT, randomized control trial; BoNT-A, botulinum toxin type A.

Study	Study Design	Region	Trial Registration No.	Population	Group	Sample Size	Gender: Female, *n* (%)	Mean Age, Years	Intervention and Comparison	Treatment Duration
Yokoyama et al. (2020) [25]	RCT	Japan	NCT02820844	OAB	Intervention	124	92 (74)	65.9	BoNT-A 100 U	12 weeks
					Control	124	94 (76)		Placebo	
Moore et al. (2015) [24]	RCT	UK/USA	NCT00910520 NCT0091845	OAB	Intervention	245	-	>65	BoNT-A 100 U	12 weeks
					Control	225	-		Placebo	
Flynn et al. (2009) [26]	RCT	USA	NCT00178191	OAB	Intervention	15	-	66	BoNT-A 200 U or 300 U	6 weeks
					Control	7	-		Placebo	
Brubaker et al. (2008) [27]	RCT	USA	NCT00373789	OAB	Intervention	28	100%	66.3	BoNT-A 200 U	4 weeks
					Control	15	100%		Placebo	

-, information regarding the gender of the patients was not available in the publication.

**Table 2:** Risk of bias assessment.

First Author	Randomization Process	Intervention Adherence	Missing Outcome Data	Outcome Measurement	Selective Reporting	Overall RoB
Yokoyama et al. (2020) [25]	S ^1^	L	S ^2^	L	L	H
Moore et al. (2015) [24]	S ^1^	L	S ^2^	L	L	H
Flynn et al. (2009) [26]	L	L	L	L	L	L
Brubaker et al. (2008) [27]	S ^1^	L	S ^3^	L	S ^4^	H

RoB, risk of bias; L, low risk of bias; S, some concerns; H, high risk of bias. ^1^ The studies did not provide allocation concealment details. ^2^ The studies did not provide explicit numbers or reasons for missing data, and the handling method was unclear. ^3^ Six subjects withdrew due to severe adverse events, including non-urinary infection and cardiovascular, neurological, and musculoskeletal system injuries. One subject in the placebo group died due to unrelated congestive heart failure. ^4^ Some secondary outcomes were not sufficiently analyzed due to an inadequate sample size.

## Data Availability

The original contributions presented in this study are included in the article/Supplementary Materials. Further inquiries can be directed to the corresponding authors.

## Supplementary Materials

The following supporting information can be downloaded at: https://www.mdpi.com/article/10.3390/toxins16110484/s1, Table S1: Search strategy.

## References

[1] Leron E., Weintraub A.Y., Mastrolia S.A., Schwarzman P. (2018). Overactive bladder syndrome: Evaluation and management. Curr. Urol..

[2] Drake M.J. (2014). Do we need a new definition of the overactive bladder syndrome? ICI-RS 2013. Neurourol. Urodyn..

[3] En M., Lin W.-Y., Lee W.-C., Chuang Y.C. (2012). Pathophysiology of overactive bladder. LUTS Low. Urin. Tract Symptoms.

[4] Przydacz M., Gasowski J., Grodzicki T., Chlosta P. (2023). Lower Urinary Tract Symptoms and Overactive Bladder in a Large Cohort of Older Poles-A Representative Tele-Survey. J. Clin. Med..

[5] Irwin D.E., Milsom I., Hunskaar S., Reilly K., Kopp Z., Herschorn S., Coyne K., Kelleher C., Hampel C., Artibani W. (2006). Population-Based Survey of Urinary Incontinence, Overactive Bladder, and Other Lower Urinary Tract Symptoms in Five Countries: Results of the EPIC Study. Eur. Urol..

[6] Gormley E.A., Lightner D.J., Faraday M., Vasavada S.P. (2015). Diagnosis and treatment of overactive bladder (non-neurogenic) in adults: AUA/SUFU guideline amendment. J. Urol..

[7] Chen L.-C., Kuo H.-C. (2019). Pathophysiology of refractory overactive bladder. LUTS Low. Urin. Tract Symptoms.

[8] Hampel C., Betz D., Burger M., Nowak C., Vogel M. (2017). Solifenacin in the elderly: Results of an observational study measuring efficacy, tolerability and cognitive effects. Urol. Int..

[9] Wagg A., Arumi D., Herschorn S., Angulo Cuesta J., Haab F., Ntanios F., Carlsson M., Oelke M. (2017). A pooled analysis of the efficacy of fesoterodine for the treatment of overactive bladder, and the relationship between safety, co-morbidity and polypharmacy in patients aged 65 years or older. Age Ageing.

[10] Cai X., Campbell N., Khan B., Callahan C., Boustani M. (2013). Long-term anticholinergic use and the aging brain. Alzheimer’s Dement..

[11] Gray S.L., Anderson M.L., Dublin S., Hanlon J.T., Hubbard R., Walker R., Yu O., Crane P.K., Larson E.B. (2015). Cumulative use of strong anticholinergics and incident dementia: A prospective cohort study. JAMA Intern. Med..

[12] Chancellor M.B., Lucioni A., Staskin D. (2024). Oxybutynin-associated Cognitive Impairment: Evidence and Implications for Overactive Bladder Treatment. Urology.

[13] Herschorn S., Staskin D., Schermer C.R., Kristy R.M., Wagg A. (2020). Safety and tolerability results from the PILLAR study: A phase iv, double-blind, randomized, placebo-controlled study of Mirabegron in patients ≥ 65 years with overactive bladder-wet. Drugs Aging.

[14] Varano S., Staskin D., Frankel J., Shortino D., Jankowich R., Mudd P.N. (2021). Efficacy and safety of once-daily vibegron for treatment of overactive bladder in patients aged ≥ 65 and ≥ 75 years: Subpopulation analysis from the EMPOWUR randomized, international, phase III study. Drugs Aging.

[15] Kalsi V., Apostolidis A., Popat R., Gonzales G., Fowler C.J., Dasgupta P. (2006). Quality of life changes in patients with neurogenic versus idiopathic detrusor overactivity after intradetrusor injections of botulinum neurotoxin type A and correlations with lower urinary tract symptoms and urodynamic changes. Eur. Urol..

[16] Aoki K.R. (2001). Pharmacology and immunology of botulinum toxin serotypes. J. Neurol..

[17] Marcelissen T., Rahnama’i M., Snijkers A., Schurch B., De Vries P. (2017). Long-term follow-up of intravesical botulinum toxin-A injections in women with idiopathic overactive bladder symptoms. World J. Urol..

[18] Kao Y.-L., Ou Y.-C., Kuo H.-C. (2022). Bladder dysfunction in older adults: The botulinum toxin option. Drugs Aging.

[19] Apostolidis A., Dasgupta P., Fowler C.J. (2006). Proposed mechanism for the efficacy of injected botulinum toxin in the treatment of human detrusor overactivity. Eur. Urol..

[20] Drake M.J. (2008). Mechanisms of action of intravesical botulinum treatment in refractory detrusor overactivity. BJU Int..

[21] Lin Y.-H., Chiang B.-J., Liao C.-H. (2020). Mechanism of action of botulinum toxin A in treatment of functional urological disorders. Toxins.

[22] Drake M.J., Nitti V.W., Ginsberg D.A., Brucker B.M., Hepp Z., McCool R., Glanville J.M., Fleetwood K., James D., Chapple C.R. (2017). Comparative assessment of the efficacy of onabotulinumtoxinA and oral therapies (anticholinergics and mirabegron) for overactive bladder: A systematic review and network meta-analysis. BJU Int..

[23] White W.M., Pickens R.B., Doggweiler R., Klein F.A. (2008). Short-term efficacy of botulinum toxin a for refractory overactive bladder in the elderly population. J. Urol..

[24] Moore C., Kaufmann A., Joshi M., Zheng Y., Herschorn S. (2015). Onabotulinumtoxina has a positive safety and efficacy profile in overactive bladder (OAB) patients <65 and ≥65 years of age. Neurourol. Urodyn..

[25] Yokoyama O., Honda M., Yamanishi T., Sekiguchi Y., Fujii K., Nakayama T., Mogi T. (2020). OnabotulinumtoxinA (botulinum toxin type A) for the treatment of Japanese patients with overactive bladder and urinary incontinence: Results of single-dose treatment from a phase III, randomized, double-blind, placebo-controlled trial (interim analysis). Int. J. Urol..

[26] Flynn M.K., Amundsen C.L., Perevich M., Liu F., Webster G.D. (2009). Outcome of a randomized, double-blind, placebo controlled trial of botulinum A toxin for refractory overactive bladder. J. Urol..

[27] Brubaker L., Richter H.E., Visco A., Mahajan S., Nygaard I., Braun T.M., Barber M.D., Menefee S., Schaffer J., Weber A.M. (2008). Refractory idiopathic urge urinary incontinence and botulinum A injection. J. Urol..

[28] Anger J.T., Weinberg A., Suttorp M.J., Litwin M.S., Shekelle P.G. (2010). Outcomes of intravesical botulinum toxin for idiopathic overactive bladder symptoms: A systematic review of the literature. J. Urol..

[29] Duthie J.B., Vincent M., Herbison G.P., Wilson D.I., Wilson D. (2011). Botulinum toxin injections for adults with overactive bladder syndrome. Cochrane Database Syst. Rev..

[30] Henriet B., Roumeguere T. (2015). Botulinum toxin injection for refractory non-neurogenic overactive bladder. Systematic review. Rev. Medicale Brux..

[31] Cui Y., Wang L., Liu L., Zeng F., Niu J., Qi L., Chen H. (2013). Botulinum toxin-A injections for idiopathic overactive bladder: A systematic review and meta-analysis. Urol. Int..

[32] Suskind A.M. (2017). The aging overactive bladder: A review of aging-related changes from the brain to the bladder. Curr. Bladder Dysfunct. Rep..

[33] Ou Y.-C., Kao Y.-L., Ho Y.-H., Wu K.-Y., Kuo H.-C. (2023). Intravesical Injection of Botulinum Toxin Type A in Patients with Refractory Overactive Bladder—Results between Young and Elderly Populations, and Factors Associated with Unfavorable Outcomes. Toxins.

[34] Chen J.-L., Kuo H.-C. (2020). Clinical application of intravesical botulinum toxin type A for overactive bladder and interstitial cystitis. Investig. Clin. Urol..

[35] Loh K., Sivalingam N. (2006). Urinary incontinence in the elderly population. Med. J. Malays..

[36] Alshammari S., Alyahya M.A., Allhidan R.S., Assiry G.A., AlMuzini H.R., AlSalman M.A. (2020). Effect of urinary incontinence on the quality of life of older adults in Riyadh: Medical and sociocultural perspectives. Cureus.

[37] Dowson C., Watkins J., Khan M.S., Dasgupta P., Sahai A. (2012). Repeated botulinum toxin type A injections for refractory overactive bladder: Medium-term outcomes, safety profile, and discontinuation rates. Eur. Urol..

[38] Craciun M., Irwin P.P. (2019). Outcomes for intravesical abobotulinumtoxin A (Dysport) treatment in the active management of overactive bladder symptoms—A prospective study. Urology.

[39] Basin M.F., Chadha P., Useva A., Ginzburg N., Ferry E. (2024). Investigation of intradetrusor onabotulinum toxin A efficacy and safety in older adults with urge urinary incontinence. Int. Urol. Nephrol..

[40] Moga M.A., Banciu S., Dimienescu O., Bigiu N.-F., Scarneciu I. (2015). Botulinum-A Toxin’s efficacy in the treatment of idiopathic overactive bladder. J. Pak. Med. Assoc..

[41] Dmochowski R., Chapple C., Nitti V.W., Chancellor M., Everaert K., Thompson C., Daniell G., Zhou J., Haag-Molkenteller C. (2010). Efficacy and safety of onabotulinumtoxinA for idiopathic overactive bladder: A double-blind, placebo controlled, randomized, dose ranging trial. J. Urol..

[42] Nitti V.W., Dmochowski R., Herschorn S., Sand P., Thompson C., Nardo C., Yan X., Haag-Molkenteller C., Group E.S. (2013). OnabotulinumtoxinA for the treatment of patients with overactive bladder and urinary incontinence: Results of a phase 3, randomized, placebo controlled trial. J. Urol..

[43] Liao C.-H., Kuo H.-C. (2013). Increased risk of large post-void residual urine and decreased long-term success rate after intravesical onabotulinumtoxinA injection for refractory idiopathic detrusor overactivity. J. Urol..

[44] Mateu Arrom L., Mayordomo Ferrer O., Sabiote Rubio L., Gutierrez Ruiz C., Martínez Barea V., Palou Redorta J., Errando Smet C. (2020). Treatment response and complications after intradetrusor onabotulinumtoxinA injection in male patients with idiopathic overactive bladder syndrome. J. Urol..

[45] Wang C.-C., Jiang Y.-H., Kuo H.-C. (2020). The pharmacological mechanism of diabetes mellitus-associated overactive bladder and its treatment with botulinum toxin A. Toxins.

[46] Wang C.C., Liao C.H., Kuo H.C. (2014). Diabetes mellitus does not affect the efficacy and safety of intravesical onabotulinumtoxinA injection in patients with refractory detrusor overactivity. Neurourol. Urodyn..

[47] Kuo H.-C., Liao C.-H., Chung S.-D. (2010). Adverse events of intravesical botulinum toxin a injections for idiopathic detrusor overactivity: Risk factors and influence on treatment outcome. Eur. Urol..

[48] Kuo H.-C. (2022). Clinical application of botulinum neurotoxin in lower-urinary-tract diseases and dysfunctions: Where are we now and what more can we do?. Toxins.

[49] Truzzi J.C., Lapitan M.C., Truzzi N.C., Iacovelli V., Averbeck M.A. (2022). Botulinum toxin for treating overactive bladder in men: A systematic review. Neurourol. Urodyn..

[50] Lee P.J., Kuo H.C. (2020). High incidence of lower urinary tract dysfunction in women with recurrent urinary tract infections. LUTS Low. Urin. Tract Symptoms.

[51] Seki N., Masuda K., Kinukawa N., Senoh K., Naito S. (2004). Risk factors for febrile urinary tract infection in children with myelodysplasia treated by clean intermittent catheterization. Int. J. Urol..

[52] Kuo Y.-C., Kuo H.-C. (2016). Adverse events of intravesical onabotulinumtoxina injection between patients with overactive bladder and interstitial cystitis—Different mechanisms of action of botox on bladder dysfunction?. Toxins.

[53] Vasudeva P., Madersbacher H. (2014). Factors implicated in pathogenesis of urinary tract infections in neurogenic bladders: Some revered, few forgotten, others ignored. Neurourol. Urodyn..

[54] Wang C.-C., Chou E.C.-L., Chuang Y.-C., Lin C.-C., Hsu Y.-C., Liao C.-H., Kuo H.-C. (2021). Effectiveness and safety of intradetrusor onabotulinumtoxina injection for neurogenic detrusor overactivity and overactive bladder patients in Taiwan—A phase IV prospective, interventional, multiple-center study (Restore study). Toxins.

[55] Prakash N.S., Lopategui D.M., Gomez C. (2017). Changes in management of poorly compliant bladder in botulinum toxin a era. Curr. Urol. Rep..

[56] Metcalf T.U., Cubas R.A., Ghneim K., Cartwright M.J., Grevenynghe J.V., Richner J.M., Olagnier D.P., Wilkinson P.A., Cameron M.J., Park B.S. (2015). Global analyses revealed age-related alterations in innate immune responses after stimulation of pathogen recognition receptors. Aging Cell.

[57] Espi M., Koppe L., Fouque D., Thaunat O. (2020). Chronic kidney disease-associated immune dysfunctions: Impact of protein-bound uremic retention solutes on immune cells. Toxins.

[58] Daryabor G., Atashzar M.R., Kabelitz D., Meri S., Kalantar K. (2020). The effects of type 2 diabetes mellitus on organ metabolism and the immune system. Front. Immunol..

[59] de Rijk M.M., Wolf-Johnston A., Kullmann A.F., Taiclet S., Kanai A.J., Shiva S., Birder L.A. (2022). Aging-associated changes in oxidative stress negatively impacts the urinary bladder urothelium. Int. Neurourol. J..

[60] Kuo H.-C. (2016). OnabotulinumtoxinA treatment for overactive bladder in the elderly: Practical points and future prospects. Drugs Aging.

[61] Gong Q.-Q., Xu Y.-Q., Xu J., Ding X.-Y., Guo C. (2020). Meta-Analysis of Randomized Controlled Trials Using Botulinum Toxin A at Different Dosages for Urinary Incontinence in Patients With Overactive Bladder. Front. Pharmacol..

[62] El Issaoui M., Elissaoui S., Elmelund M., Klarskov N. (2023). Bleeding risk in female patients undergoing intravesical injection of onabotulinumtoxinA for overactive bladder: A Danish retrospective cohort study. Int. Urogynecol. J..

[63] Page M.J., McKenzie J.E., Bossuyt P.M., Boutron I., Hoffmann T.C., Mulrow C.D., Shamseer L., Tetzlaff J.M., Akl E.A., Brennan S.E. (2021). The PRISMA 2020 statement: An updated guideline for reporting systematic reviews. BMJ.

[64] Kang H. (2021). Trial sequential analysis: Novel approach for meta-analysis. Anesth. Pain Med..

