# Supplementary material for: Evaluating the Efficacy and Safety of Botulinum Toxin in Treating Overactive Bladder in the Elderly: A Meta-Analysis with Trial Sequential Analysis of Randomized Controlled Trials
Source: Toxins (Basel). 2024 Nov 8;16(11):484. doi: 10.3390/toxins16110484 (PMC11597992; doi:10.3390/toxins16110484)
Supplement: Supplementary file 1 [file toxins-16-00484-s001.zip › Table S1_Search strategy.pdf]

Table S1. Search Strategy

Database	#	Search syntax	Citations found
1) Embase	1	((Botulinum NEAR/2 (toxin OR neurotoxin)) OR "BTX" OR "BoNT" OR "Botox"):ti,ab,kw,de	51326
	2	"botulinum toxin A"/exp	28366
	3	("Urgency-frequency syndrome*" OR "detrusor-sphincteric dyssynergi*" OR OAB OR ((overactiv* OR over-activ* OR hyperactiv* OR hyper-activ* OR hyperreflexi* OR hyper-reflexi* OR instabilit* OR urge*) NEAR/4 (bladder* OR urinary OR Detrusor* OR incontinen*))):ti,ab,kw,de	38025
	4	"Urinary dysfunction"/de OR "bladder instability"/exp OR "detrusor dyssynergia"/exp OR "neurogenic bladder"/exp OR "overactive bladder"/exp OR "urinary urgency"/exp OR "urine extravasation"/exp	44925
	5	(elder* OR old OR aging OR old-age* OR aged OR senior* OR senility):ti,ab,kw,de	7961513
	6	"aged"/de OR "aged hospital patient"/exp OR "frail elderly"/exp OR "institutionalized elderly"/exp OR "very elderly"/exp	3971773
	7	(#1 OR #2) AND (#3 OR #4) AND (#5 OR #6) AND [embase]/lim	1061
	8	(#7) AND ("randomized controlled trial"/de or "controlled clinical study"/de or "randomization"/de or "intermethod comparison"/de or "double blind procedure"/de or "human experiment"/de OR (random* or placebo or "parallel group$" or crossover or "cross over" or assigned or allocated or volunteer or volunteers):ti,ab OR (open NEAR/1 label):ti,ab OR ((double or single or doubly or singly) NEAR/1 (blind or blinded or blindly)):ti,ab OR ((assign* or match or matched or allocation) NEAR/5 (alternate or group$ or intervention$ or patient$ or subject$ or participant$)):ti,ab OR (controlled NEAR/7 (study or design or trial)):ti,ab OR (compare or compared or comparison or trial):ti OR ((evaluated or evaluate or evaluating or assessed or assess) and (compare or compared or comparing or comparison)):ab) NOT (((random* NEAR/1 sampl* NEAR/7 ("cross section*" or questionnaire$ or survey* or database$)):ti,ab not ("comparative study"/de or "controlled study"/de or "randomi$ed controlled":ti,ab or "randomly assigned":ti,ab)) OR ("cross-sectional study"/de not ("randomized controlled trial"/de or "controlled clinical study"/de or "controlled study"/de or "randomi$ed controlled":ti,ab or "control group$":ti,ab)) OR ((((case NEAR/1 control*) and random*) not "randomi$ed controlled"):ti,ab) OR (("systematic review" not (trial or study)):ti) OR ((nonrandom* not random*):ti,ab) OR ("random field*":ti,ab) OR (("random cluster" NEAR/3 sampl*):ti,ab) OR ((review:ab and review/it) not trial:ti) OR ("we searched":ab and (review:ti or review/it)) OR ("update review":ab) OR ((databases NEAR/4 searched):ab) OR ((rat or rats or mouse or mice or swine or porcine or murine or sheep or lambs or pigs or piglets or rabbit or rabbits or cat or cats or dog or dogs or cattle or bovine or monkey or monkeys or trout or marmoset*):ti and "animal experiment"/de) OR ("animal experiment"/de not ("human experiment"/de or "human"/de)))	340

2) Pubmed (OvidMedline)	1	"Botulinum toxin"[tiab:~1] OR Botox [tiab] OR "Botulinum neurotoxin"[tiab:~1] OR BTX[tiab] OR BoNT[tiab]	22573
	2	botulinum toxins [mh]	19058
	3	("Urgency-frequency syndrome*" OR "detrusor-sphincteric dyssynergi*" OR OAB OR ((overactiv* OR over-activ* OR hyperactiv* OR hyper-activ* OR hyperreflexi* OR hyper-reflexi* OR instabilit* OR urge*) ADJ4 (bladder* OR urinary OR Detrusor* OR incontinen*))).mp	14250
	4	Urinary Bladder, Neurogenic [mh] OR Urinary Bladder, Overactive [mh]	13667
	5	elder*[tiab] OR old[tiab] OR aging[tiab] OR old-age[tiab] OR aged[tiab] OR senior[tiab] OR senility[tiab]	2422425
	6	aged[MeSH Terms]	3490208
	7	(#1 OR #2) AND (#3 OR #4) AND (#5 OR #6)	380
	8	(#7) AND (randomized controlled trial[pt] OR controlled clinical trial[pt] OR randomized[tiab] OR randomised[tiab] OR placebo[tiab] OR drug therapy[sh] OR randomly[tiab] OR trial[tiab] OR groups[tiab] NOT (animals [mh] NOT humans [mh]))	314
3) Cochrane CENTRAL	1	((Botulinum NEAR/1 (toxin OR neurotoxin)) OR "BTX" OR "BoNT" OR "Botox"):ti,ab,kw	4381
	2	[mh "botulinum toxins"]	2574
	3	("Overactive bladder" OR OAB OR "Urge incontinence" OR (Detrusor NEXT overactiv*) OR (Bladder NEXT hyperactiv*) OR (Bladder NEXT urgen*) OR "Bladder instability" OR "Urinary urgency" OR "Urgency-frequency syndrome" OR "neurogenic detrusor overactivity" OR "detrusor hyperreflexia" OR "detrusor-sphincteric dyssynergia"):ti,ab,kw	4768
	4	[mh "Urinary Bladder, Neurogenic"] OR [mh "Urinary Bladder, Overactive"]	1482
	5	(elder* OR old OR aging OR old-age* OR aged OR senior* OR senility):ti,ab,kw	735552
	6	[mh "aged"]	274545
	7	(#1 OR #2) AND (#3 OR #4) AND (#5 OR #6)	145
4) CINAHL (EBSCOhost)	1	(Botulinum N2 (toxin OR neurotoxin)) OR BTX OR BoNT OR Botox	7434
	2	mh "Botulinum Toxins+"	5962
	3	"Urgency-frequency syndrome*" OR "detrusor-sphincteric dyssynergi*" OR OAB OR ((overactiv* OR over-activ* OR hyperactiv* OR hyper-activ* OR hyperreflexi* OR hyper-reflexi* OR instabilit* OR urge*) N3	4141

		(bladder* OR urinary OR Detrusor* OR incontinen*))	
	4	mh "Overactive bladder+" OR mh "Bladder, Neurogenic+" OR mh "Urge Incontinence+"	3946
	5	elder* OR old OR aging OR old-age* OR aged OR senior* OR senility	1281099
	6	mh "Aged+" OR mh "Hospitalization of Older Persons+" OR mh "Frail Elderly+"	957357
	7	(#1 OR #2) AND (#3 OR #4) AND (#5 OR #6)	81
	8	#7 AND (MH ("randomized controlled trials" OR "double-blind studies" OR "single-blind studies" OR "random assignment" OR "pretest-posttest design" OR "cluster sample") OR TI (randomised OR randomized) OR AB (random*) OR TI (trial) OR (MH (sample size) AND AB (assigned OR allocated OR control)) OR MH (placebos) OR PT (randomized controlled trial) OR AB (control W5 group) OR MH ("crossover design" OR "comparative studies") OR AB (cluster W3 RCT)) NOT ((MH ("animals+" OR "animal studies") OR TI (animal model*)) NOT MH (human))	25
5) Scopus	1	title-abs ((Botulinum W/1 (toxin OR neurotoxin)) OR "BTX" OR "BoNT" OR "Botox") OR authkey ((Botulinum W/1 (toxin OR neurotoxin)) OR "BTX" OR "BoNT" OR "Botox")	30376
	2	title-abs ("Overactive bladder" OR OAB OR "Urge incontinence" OR "Detrusor overactiv*" OR "Bladder hyperactiv*" OR "Bladder urgen*" OR "Bladder instability" OR "Urinary urgency" OR "Urgency-frequency syndrome" OR "neurogenic detrusor overactivity" OR "detrusor hyperreflexia" OR "detrusor-sphincteric dyssynergia") OR authkey ("Overactive bladder" OR OAB OR "Urge incontinence" OR "Detrusor overactiv*" OR "Bladder hyperactiv*" OR "Bladder urgen*" OR "Bladder instability" OR "Urinary urgency" OR "Urgency-frequency syndrome" OR "neurogenic detrusor overactivity" OR "detrusor hyperreflexia" OR "detrusor-sphincteric dyssynergia")	17756
	3	title-abs (elder* OR old OR aging OR old-age* OR aged OR senior* OR senility) OR authkey (elder* OR old OR aging OR old-age* OR aged OR senior* OR senility)	3874765
	4	INDEXTERMS ("clinical trials" OR "clinical trials as a topic" OR "randomized controlled trial" OR "Randomized Controlled Trials as Topic" OR "controlled clinical trial" OR "Controlled Clinical Trials" OR "random allocation" OR "Double-Blind Method" OR "Single-Blind Method" OR "Cross-Over Studies" OR "Placebos" OR "multicenter study" OR "double blind procedure" OR "single blind procedure" OR "crossover procedure" OR "clinical trial" OR "controlled study" OR "randomization" OR "placebo") OR TITLE-ABS-KEY ("clinical trials" OR "clinical trials as a topic" OR "randomized controlled trial" OR "Randomized Controlled	

		Trials as Topic" OR "controlled clinical trial" OR "Controlled Clinical Trials as Topic" OR "random allocation" OR "randomly allocated" OR "allocated randomly" OR "Double-Blind Method" OR "Single-Blind Method" OR "Cross-Over Studies" OR "Placebos" OR "cross-over trial" OR "single blind" OR "double blind" OR "factorial design" OR "factorial trial") OR TITLE (clinical trial OR trial OR rct* OR random* OR blind*)	
	5	#1 AND #2 AND #3 AND #4	52
